# Comment on “Improving the efficiency of a CIGS solar cell to above 31% with Sb_2_S_3_ as a new BSF: a numerical simulation approach by SCAPS-1D” by M. F. Rahman and S. Goumri-Said *et al.*, *RSC Adv.*, 2024, **14**, 1924

**DOI:** 10.1039/d4ra03002h

**Published:** 2024-10-07

**Authors:** Alexander P. Kirk

**Affiliations:** a Central R&D LLC Schaumburg IL 60194 USA apkirk88@gmail.com

## Abstract

It was reported in early 2024 that a single-junction 1.1 eV bandgap copper indium gallium selenide (CIGS) solar cell can achieve actual power conversion efficiency up to 40.70%, open circuit voltage up to 1.330 V, and fill factor up to 90.55% at 300 K when the solar cell is irradiated by the air mass 1.5 global (AM1.5G) solar spectrum (M. F. Rahman *et al.*, *RSC Adv.*, 2024, **14**, 1924–1938). These simulated solar cell performance parameters exceed the ideal detailed balance-limiting power conversion efficiency, open circuit voltage, and fill factor of a 1.1 eV bandgap single-junction solar cell.

## Abstract 2

In this Comment, the detailed balance-limiting open circuit voltage, fill factor, and power conversion efficiency are calculated for a 1.1 eV bandgap copper indium gallium selenide (CIGS) solar cell at 300 K when irradiated by the air mass 1.5 global (AM1.5G) spectrum.

## 1 Introduction

**Fig. 1:**
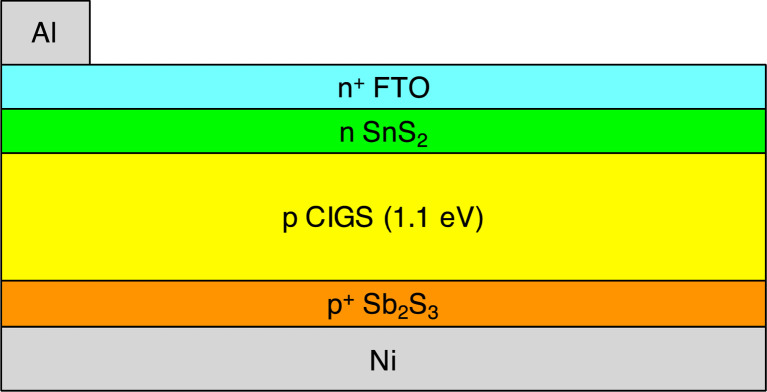
Cross section view of 1.1 eV bandgap CIGS solar cell.

Based on simulations run with a conventional one-dimensional drift-diffusion solar cell modeling program, SCAPS-1D, Rahman *et al.* reported that a conventional 1.1 eV bandgap copper indium gallium selenide (CIGS) solar cell can achieve actual air mass 1.5 global (AM1.5G) power conversion efficiency *η* up to 40.70%, open circuit voltage *V*_oc_ up to 1.330 V, and fill factor FF up to 90.55%.^1^ The device design that was simulated contains an aluminum (Al) top grid contact, a 50 nm thick fluorine-doped tin oxide (FTO) layer, a 50 nm thick tin disulfide (SnS_2_) layer, a 1.1 eV bandgap CIGS primary absorbing layer with thickness up to 3000 nm, a 200 nm thick antimony trisulfide (Sb_2_S_3_) layer, and a nickel (Ni) rear contact. A schematic of the solar cell proposed by Rahman *et al.* is shown in Fig. 1.

## 2 Discussion

**Fig. 2:**
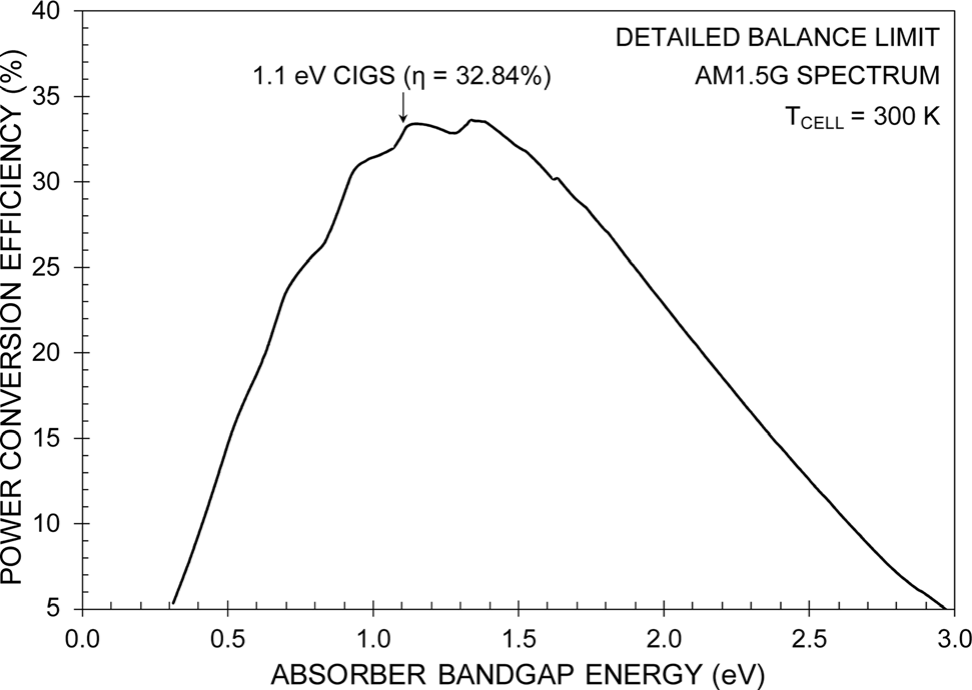
Detailed balance-limiting power conversion efficiency.

Before modeling any type of solar cell with a standard drift-diffusion computer program, it is helpful to determine its theoretical efficiency limit. In the field of photovoltaics, this is typically accomplished by using the principle of detailed balance to calculate the ideal limiting efficiency.^2,3^ The benefit of this approach is that the detailed balance formalism allows for a fundamental determination of the efficiency limit of a solar cell independent of specific device design features such as the thickness of individual layers, the dopant concentrations, and the contact metal work functions. Moreover, the only sink for photogenerated electron and hole recombination at steady-state and open circuit is the necessary radiative recombination whereas the undesirable nonradiative Auger and Shockley–Read–Hall recombination are not considered. A plot of the detailed balance-limiting power conversion efficiency *η* of single-junction solar cells, as a function of absorber bandgap energy, is shown in Fig. 2.

The equations required to calculate the detailed balance-limiting performance are listed next. The open circuit voltage is expressed as *V*_oc_ = *E*_g_/*q* − (*kT*/*q*)ln[(2π*qE*_g_^2^*kT*)/(*h*^3^*c*^2^*J*_sc_)], where *E*_g_ is the bandgap energy, *q* is the electron charge, *k* is the Boltzmann constant, *T* is the cell temperature, *h* is the Planck constant, and *c* is the speed of light in vacuum. The max power point voltage is expressed as *V*_mp_ ≈ *V*_oc_ − (*kT*/*q*)ln[1 + (*qV*_oc_/*kT*)]. The max power point current density is expressed as *J*_mp_ = *J*_sc_/[1 + (*kT*/*qV*_mp_)]. The fill factor is expressed as FF = (*J*_mp_*V*_mp_)/(*J*_sc_*V*_oc_). The power conversion efficiency is expressed as *η* = *P*_out_/*P*_in_, where *P*_out_ = *J*_mp_*V*_mp_ and *P*_in_ = 0.1 W cm^−2^ (AM1.5G irradiance).

The maximum available short circuit current density *J*_sc_ of a 1.1 eV bandgap semiconductor is 44.23 mA cm^−2^ when considering the AM1.5G solar spectrum. In Table 1, the detailed balance-limiting *V*_oc_, FF, and *η* are shown for two cases. In Case 1, the maximum AM1.5G spectrum *J*_sc_ of 44.23 mA cm^−2^ is considered. In Case 2, a reduced *J*_sc_ of 34.55 mA cm^−2^ is considered. This smaller *J*_sc_ value was chosen to match the peak *J*_sc_ reported by Rahman *et al.*^1^ Nonetheless, in both cases the detailed balance-limiting *V*_oc_, FF, and *η* are less than the peak values reported by Rahman *et al.*^1^ In particular, refer to the simulated *V*_oc_, FF, and *η* values reported in Section 4.3 on p. 1932 as well as Fig. 4 on p. 1929, Fig. 6 on p. 1931, and Fig. 7 on p. 1932 of the article by Rahman *et al.*^1^ Not only do these figures show simulated peak *V*_oc_, FF, and *η* exceeding the ideal detailed balance limit, they also show that these simulated peak performance parameters occur when the bulk and interface defect concentrations are the largest of the range of values that were simulated by Rahman *et al.*^1^ Note, too, that the peak *V*_oc_ (1.330 V) reported by Rahman *et al.* exceeds the bandgap-equivalent voltage (*E*_g_/*q* = 1.1 V) of the CIGS absorber.

**Table 1:** Detailed balance-limiting performance of a 1.1 eV CIGS solar cell irradiated by the AM1.5G spectrum with *T*_cell_ = 300 K^a^

Case	Absorber	*E*_g_ (eV)	*J*_sc_ (mA cm^−2^)	*V*_oc_ (V)	*V*_mp_ (V)	*J*_mp_ (mA cm^−2^)	FF (%)	*η* (%)
1	CIGS	1.1	44.23	0.8590	0.7676	42.79	86.46	32.84
2	CIGS	1.1	34.55	0.8526	0.7614	33.42	86.38	25.44

a Key: *E*_g_ is bandgap energy, *J*_sc_ is short circuit current density, *V*_oc_ is open circuit voltage, *V*_mp_ is max power point voltage, *J*_mp_ is max power point current density, FF is fill factor, and *η* is power conversion efficiency. Note: “Case 1” assumes maximum AM1.5G *J*_sc_ whereas “Case 2” assumes a reduced *J*_sc_. Note: Rahman *et al.* reported *V*_oc_ up to 1.330 V, FF up to 90.55%, and *η* up to 40.70%.

## 3 Conclusion

In summary, Rahman *et al.* reported unrealistic values of open circuit voltage *V*_oc_, fill factor FF, and power conversion efficiency *η* of a 1.1 eV CIGS single-junction solar cell even though their proposed solar cell is a conventional device that was modeled with standard drift-diffusion physics. More to the point, in their device simulations, Rahman *et al.* did not consider hot carrier collection or some other attribute that might result in actual open circuit voltage and power conversion efficiency exceeding the detailed balance-limiting open circuit voltage and power conversion efficiency.

## Author contributions

Alexander P. Kirk: conceptualization, formal analysis, visualization, writing – original draft, review & editing.

## Conflicts of interest

There are no conflicts of interest to declare.
